# Insights from Three Pan-European Multicentre Studies on Invasive *Candida* Infections and Outlook to ECMM *Candida* IV

**DOI:** 10.1007/s11046-024-00871-0

**Published:** 2024-08-01

**Authors:** Stella Wolfgruber, Sarah Sedik, Lena Klingspor, Annamaria Tortorano, Neil A. R. Gow, Katrien Lagrou, Jean-Pierre Gangneux, Johan Maertens, Jacques F. Meis, Cornelia Lass-Flörl, Sevtap Arikan-Akdagli, Oliver A. Cornely, Martin Hoenigl

**Affiliations:** 1 Division of Infectious Diseases, Department of Internal Medicine, Medical University of Graz, Auenbruggerplatz 15, 8036 Graz, Austria; 2 Translational Medical Mycology Research Unit, ECMM Excellence Center for Medical Mycology, Medical University of Graz, Graz, Austria; 3 BioTechMed-Graz, Graz, Austria; 4 Division of Clinical Microbiology, Department of Laboratory Medicine, Karolinska Institutet, Stockholm, Sweden; 5 Dipartimento Scienze Biomediche per la Salute, Università degli Studi di Milano, Milan, Italy; 6 Medical Research Council Centre for Medical Mycology, Department of Biosciences, University of Exeter, Geoffrey Pope Building, Stocker Road, Exeter, UK; 7 Laboratory of Clinical Microbiology, Department of Microbiology, Immunology and Transplantation, KU Leuven, Louvain, Belgium; 8 Department of Laboratory Medicine and National Reference Center for Mycosis, UZ Leuven, Leuven, Belgium; 9 Univ Rennes, CHU Rennes, Inserm, EHESP, Irset (Institut de recherche en santé, environnement et travail), UMR_S 1085, 35000 Rennes, France; 10 Laboratory of Mycology, Centre Hospitalier Universitaire de Rennes, Centre National de référence pour les mycoses et antifongiques – LA AspC, ECMM Excellence Center for Medical Mycology, Rennes, France; 11 Department of Haematology and ECMM Excellence Center for Medical Mycology, University Hospitals Leuven, Campus Gasthuisberg, Leuven, Belgium; 12 Department of Medical Microbiology, Excellence Center for Medical Mycology (ECMM), Center of Expertise in Mycology Radboudumc/CWZ, Nijmegen, The Netherlands; 13 Institute of Hygiene and Medical Microbiology Medical University of Innsbruck, Excellence Center for Medical Mycology (ECMM), Innsbruck, Austria; 14 Department of Medical Microbiology, Hacettepe University Medical School, Ankara, Turkey; 15 University of Cologne, Faculty of Medicine and University Hospital Cologne, Department I of Internal Medicine, Center for Integrated Oncology Aachen Bonn Cologne Duesseldorf (CIO ABCD) and Excellence Center for Medical Mycology (ECMM), Cologne, Germany; 16 University of Cologne, Faculty of Medicine and University Hospital Cologne, Cologne, Excellence Cluster on Cellular Stress Responses in Aging-Associated Diseases (CECAD), Cologne, Germany; 17 German Centre for Infection Research (DZIF), Partner Site Bonn-Cologne, Cologne, Germany; 18 University of Cologne, Faculty of Medicine and University Hospital Cologne, Clinical Trials Centre Cologne (ZKS Köln), Cologne, Germany

**Keywords:** *Candida*, Candidemia, European Confederation of Medical Mycology (ECMM)

## Abstract

Invasive candidiasis and candidemia remain a significant public health concern. The European Confederation of Medical Mycology (ECMM) conducted three pan-European multicentre studies from 1997 to 2022 to investigate various aspects of invasive *Candida* infections. These studies revealed shifting trends in *Candida* species distribution, with an increase of non-*albicans Candida* species as causative pathogens, increasing rates of antifungal resistance, and persistently high mortality rates. Despite advancements in antifungal treatment, the persistently high mortality rate and increasing drug resistance, as well as limited drug access in low-income countries, underscore the need for continued research and development in the treatment of *Candida* infections. This review aims to summarize the findings of the three completed ECMM *Candida* studies and emphasize the importance of continued research efforts. Additionally, it introduces the upcoming ECMM *Candida* IV study, which will focus on assessing candidemia caused by non-*albicans Candida* species, including *Candida auris*, investigating antifungal resistance and tolerance, and evaluating novel treatment modalities on a global scale.

**Supplementary Information:**

The online version contains supplementary material available at 10.1007/s11046-024-00871-0.

## Introduction

The incidence of invasive fungal infections has strongly increased over recent decades, with *Candida* species remaining the primary cause of invasive nosocomial fungal infections [1–3]. Candidemia remains the most common bloodstream fungal infection in hospitalized patients, affecting male and female individuals alike [4, 5]. Globally, candidemia accounts for approximately 700,000 cases annually [6, 7], with a cumulative incidence of 7.07 episodes per 1000 intensive care unit (ICU) admissions in Europe and a remarkably high 90-day mortality rate of 43% [7–9]. At least 15 different *Candida* species are known to cause infections in humans, but the most common species responsible for invasive infections are *C. albicans*, *C. glabrata (Nakaseomyces glabratus)*, *C. parapsilosis*, *C. tropicalis* and *C. krusei* (*Pichia kudriavzevii*) [3, 10]. While *C. albicans* remains the most common species causing infections, there has been a notable increase in *non-albicans Candida* species [8, 11]. Geographic location, patient demographics, and clinical environments influence the prevalence of these species [32, 33].

Of particular interest is the emergence of *C. auris*, which is thought to have evolved from a plant saprophyte in specialized ecosystems such as wetlands [12] and adapted to higher temperatures as a result of climate change, making it a human pathogen. The species was first documented in Asia in 2009 [13, 14] has since spread worldwide and has become a fungal pathogen of critical importance [15, 16]. Since its first description, *C. auris* infections have been reported in more than 40 countries with a high mortality rate of 30–60% [17]. Of particular concern is the increase in echinocandin-resistant *C. auris* strains, as echinocandis are used as a first-line treatment for invasive *Candida* infections, including *C. auris* [16].

Important risk factors making individuals susceptible to *Candida* infections include diabetes mellitus, advanced age, prolonged antibiotic use, haemodialysis, the presence of central-venous catheter (CVC), immunosuppressive therapy, invasive procedures, prolonged hospital stay or ICU admission, mechanical ventilation, total parenteral nutrition, transplantation and chemotherapy [5, 11, 18].

To investigate epidemiology, resistance, and other aspects of invasive candidiasis (IC) the European Confederation of Medical Mycology (ECMM) conducted three pan-European multicentre studies between 1997 and 2022 [19–21].

Before 1997, most data on *Candida* bloodstream infections were collected in studies carried out in the USA. Therefore, the ECMM initiated the first multicentre study aimed to update the epidemiological and mycological profile of candidemia across Western Europe [19].

Recognizing the lack of data on surgical ICU patients the ECMM initiated the second multicentre study on IC, focusing on this subgroup [20]. In 2018, the ECMM introduced the Quality of Clinical Candidemia Management (EQUAL *Candida*) score as the first published score quantifying diagnostic and therapeutic clinical management quality [22]. The score summarizes key recommendations of international clinical guidelines for diagnosing and managing candidemia, which aim to enhance patient outcomes and survival; however, their impact has rarely been assessed. Although single-centre studies have demonstrated the EQUAL *Candida* score’s ability to predict mortality in CVC associated candidemia [23] and candidemia caused by *C. tropicalis* [24], larger multicentre assessments were lacking. To address this gap, the ECMM conducted the third pan-European multicentre study spanning from 2018 to 2019 [21]. The objective was to assess the correlation between adherence to guideline recommendations for managing candidemia and outcomes.

All three studies have provided valuable insights into *Candida* infections, including species distribution, antifungal resistance, and the treatment efficacy. This review aims to summarize the findings of these studies and underscore the importance of continued research, leading to the initiation of the *Candida* IV study. *Candida* IV will focus on non-*albicans Candida* species, evaluating antifungal resistance and tolerance on a global scale.

## Study Methodologies

Each ECMM study followed a multicentre, prospective design, enrolling patients with invasive *Candida* infections across multiple European countries within a defined observation period. The studies collected comprehensive data on patient demographics, underlying conditions, microbiological characteristics, antifungal therapy, and clinical outcomes, and were all approved by the respective ethical committees. Robust statistical analyses were conducted to assess temporal trends and identify factors associated with outcomes.

The first study, conducted from 1997 to 1999 [19], primarily focused on epidemiological data of candidemia based on hospital records. Patient data were collected over a 30-day surveillance period, with each hospital providing the first isolate obtained for each episode of candidemia. Subsequent analysis of these isolates was conducted at the participating hospitals. During this study no susceptibility testing or other testing was performed.

Building on the first initiative, the second study focused on the impact of IC, particularly in surgical intensive care patients [20]. This study aimed to understand IC characteristics, document its epidemiology, and identify factors associated with mortality. It highlighted changes in the *Candida* species causing infections and emphasized the importance of surveillance studies, particularly focusing on surgical intensive care patients, who have often been overlooked in previous studies [5–7]. *Candida* isolates were identified at species level in the laboratories of each institution using standard methods. Susceptibility testing was not performed.

The most recent study, conducted from 2018 to 2022 (cases collected during a 1-year observation period between mid-2018 and mid-2019), aimed to provide a comprehensive and representative picture of culture-proven candidemia in Europe, by limiting enrolment per country and participating hospitals according to population size [21]. This multicentre observational cohort study collected extensive data on epidemiology, risk factors, species distribution, antifungal susceptibility profiles, treatment approaches and patient outcomes from 632 participants with a median age of 65 years. The study focused on adherence to guideline recommendations and their impact on clinical outcomes [21]; in addition, antifungal susceptibility testing was performed centrally.

## Definitions

In the first study, candidemia was defined by the isolation of *Candida* species from one or more blood cultures in patients exhibiting clinically relevant signs and symptoms. Additionally, the study accounted for mucous membrane colonization, particularly if stool and/or oropharyngeal samples had been tested for *Candida* spp. within the 2 weeks preceding the candidaemic episode [19]. In the second study [20], invasive candidiasis with or without candidemia was defined as a positive culture for *Candida* obtained from blood or other sterile body sites, following the 2008 EORTC/MSG criteria [25]. The most recent study [21] defined candidemia according to 2012 ESCMID criteria [26].

## Countries Participating in Each Study

The initial study [19] focused on the epidemiology of candidemia across seven European countries: Austria, Germany, France, Italy, Spain, Sweden, and the UK. Participating hospitals were selected by national coordinators based on their capacity to comprehensively document all candidemia episodes occurring during the observation period. The coordinators played a crucial role in ensuring compliance with study protocols and data collection procedures. A total of 2,089 cases of candidemia were documented by 106 institutions during the 28-month study period.

The second ECMM survey [20] shifted its focus to exclusively investigate invasive *Candida* infections in surgical patients within European intensive care units (ICUs). Expanding its reach, the prospective study included 14 European countries with additional participating countries including Czech Republic, Finland, Germany, Greece, Hungary, the Netherlands, Portugal, and Turkey. Similar to the first study the participating centres were selected via national coordinators who collaborated with ICU colleagues to enrol a total of 779 surgical ICU patients with invasive candidosis. Clinicians and mycologists completed a standardized questionnaire for each case, with patients assigned code numbers by the national coordinator to maintain anonymity.

The third study [21] examined epidemiological data, risk factors, antifungal susceptibility profiles, treatment, and outcomes of patients with culture-proven candidemia across 64 institutions in 20 European countries. Participating hospitals were selected by ECMM council representatives of each participating country serving as national coordinators, and in addition via the EPICOVIDEHA [27], an international open web-based registry for patients with hematological malignancies infected with SARS-CoV-2 and FungiScope [28] networks and among the ECMM Global Guidelines contributor and fellow groups [29]. Enrolment in the study varied based on country size: countries with over 50 million inhabitants recruited patients from up to ten hospitals, while those with populations between 25 and 50 million recruited from up to four hospitals. Countries with populations below 25 million recruited patients from up to two hospitals each. The primary objective was to assess the association between guideline adherence and outcomes, with a secondary objective of evaluating the epidemiology, risk factors, treatment, and outcomes of candidemia patients across Europe.

**Fig. 1:**
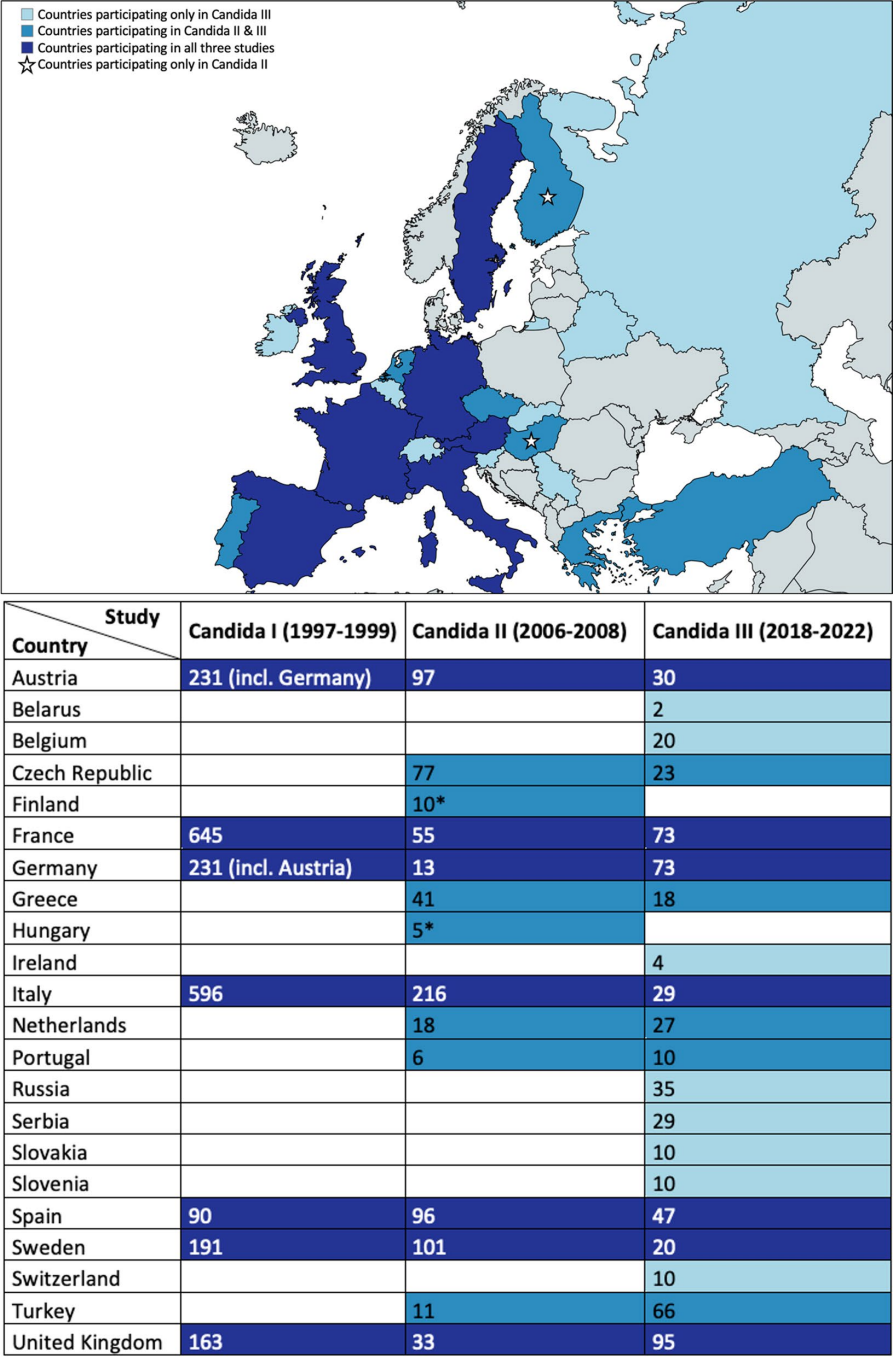
Participating European countries in the three ECMM *Candida* studies and numbers of included cases

Participating countries during the three ECMM *Candida* studies and the number of the included cases per country and study are illustrated in Fig. 1.

## Key Results of the Studies

At the time of its publication, ECMM *Candida* I was the first multicentre study worldwide. This prospective study documented 2,089 cases across 106 institutions over a 28-month period in seven European countries [19]. Although *C. albicans* was the most frequently identified pathogen, accounting for 56% of infections, the, compared to previous studies, high proportion of non-*albicans* species (43.6%) was noted [19]. Most infections occurred in younger patients, with only 28% observed in patients over 70 years old, while children under the age of one accounted for only 7.6% of cases [19].

A total of 779 patients, with a median age of 63, were enrolled from 72 ICUs across 14 European countries into ECMM *Candida* II. Among all patients enrolled, 10.8% were infected with *Candida* before ICU admission, while an additional 8.7% developed the infection within 48 h post admission. In 80.5% of surgical patients, the infections occurred more than 48 h after admission to the ICU and was then classified as ICU-acquired. In 57 participating ICUs, the median rate of candidemia was nine cases per 1000 admissions following surgery [20].

In ECMM *Candida* III independent baseline predictors of mortality included increasing age, ICU admission, high Charlson comorbidity index scores, and infection with *C. tropicalis*. The study revealed a persistently high 90-day mortality rate of 43% across Europe. Lower adherence to guidelines was linked to higher mortality rates, underlining the importance of guideline compliance in managing candidemia successfully. Additionally, the study found that first-line treatment of candidemia with echinocandins, while associated with better overall survival rates, also led to prolonged hospital stays solely for completing parenteral echinocandin treatment. This was primarily due to the lack of oral antifungal alternatives or new antifungals with a longer half-life at the time the study was conducted [30].

## *Candida* Species Distribution

**Fig. 2:**
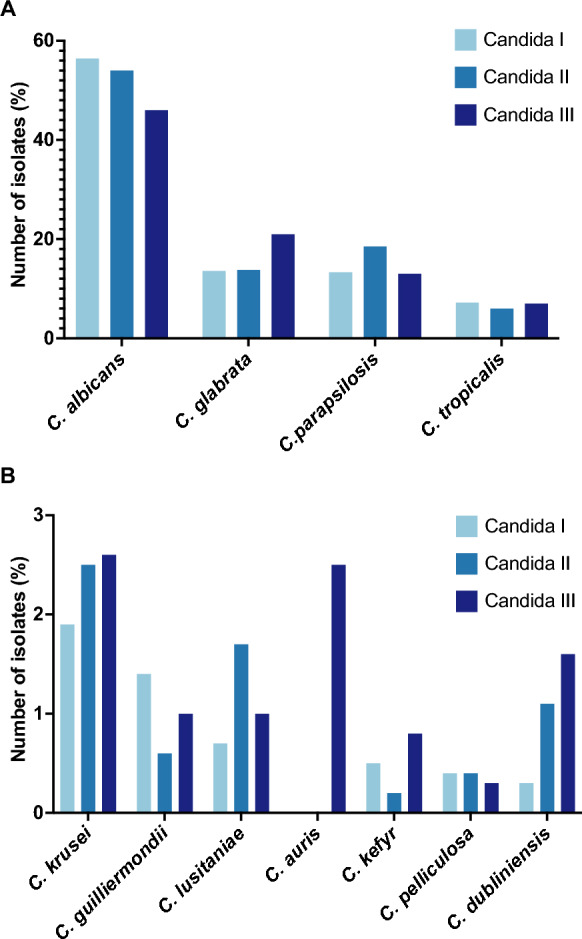
**A** Species distribution (number of common isolates) during the three ECMM *Candida* studies, **B** species distribution (number of rare isolates) during the three ECMM *Candida* studies

In recent decades, a shift from *C. albicans* as the primary pathogen to non-*albicans Candida* species has been increasingly observed. This observation was also confirmed in the three ECMM studies, as shown in Fig. 2.

The third ECMM study also evaluated antifungal resistance in candidaemia across Europe [21]. Acquired fluconazole resistance was common in *C. glabrata* (*N. glabratus*) and emerging in *C. parapsilosis*, particularly in southern Europe, indicating a concerning trend [31]. Azole resistance often involves multiple factors, e.g. target gene mutations, upregulation and efflux pumps, which usually develop over a period of months [32, 33]. Additionally, rare instances of acquired echinocandin resistance were identified in *Candida* III, including an echinocandin resistant *C. parapsilosis* isolate [31]. Resistance to echinocandins is usually associated with hotspot mutations in the target genes *fks1* and f*ks2*, especially at subtherapeutic drug levels and biofilm formation [31].

Rarely identified *Candida* species are depicted in Fig. 2B, with causative species differing based on the patients’ underlying condition.

### *Candida albica*

Across all three studies, *C. albicans* consistently continues to be the most prevalent causative agent for invasive *Candida* infections and was also the predominant species detected on catheters during *Candida* II [19–21]. However, its prevalence decreased over time, from 56.4% in the initial study [19], 54% in ECMM *Candida* II [20], and further to 46% in the most recent ECMM *Candida* III study [21]. This observation is also in line with other publications over the past decades [34, 35]. Mixed infections involving both *C. albicans* and *C. glabrata* (*N. glabratus*) were most commonly observed in the first study [19]. While *C. albicans* remained the predominant causative species, other species were frequently isolated as well, particularly among patients with haematological malignancies, underscoring the significance of non-*albicans Candida* strains [19].

In the second study, the time from ICU admission to the first positive blood culture was on average 10 days for *C. albicans* [20]. The mortality rate associated with *C. albicans* was 36.8% in the second study. Notably, the prevalence of *C. albicans* was highest in southern Europe, followed by *C. parapsilosis* and *C. glabrata (N. glabratus)* [20].

### *Candida glabrata* (*Nakaseomyces glabratus*)

*Candida glabrata* (*N. glabratus*) is of particular significance due to its intrinsic reduced susceptibility to fluconazole and genuine fluconazole resistance observed in varying rates. During the first study period, *C. glabrata (N. glabratus)* was predominantly observed in surgical patients (16.3%), ICU patients (11.9%) and those with solid tumours (15.9%) [19].

There was a notable rise in *C. glabrata* infections with increasing age, escalating from 3 to 19% [19]. In the second study, *C. glabrata* (*N. glabratus*) was isolated in 13.8% of all cases with a median ICU stay of 18 days. The time from ICU admission to the first positive blood culture was seven days, with an associated mortality rate of 43.6% [20]. *C. glabrata* (*N. glabratus*) was frequently isolated following abdominal surgery, with neurosurgical patients exhibiting lowest rates [20]. The prevalence of *C. glabrata* (*N. glabratus*) increased with age (> 60 year), reaching 71.8% [20]. The pathogen was more commonly found in northern Europe [20]. In the third study, 12% of *C. glabrata* (*N. glabratus*) isolates exhibited fluconazole resistance, with the highest rates observed in Belgium, Czech Republic, Italy, Sweden, Turkey, and the UK. Notably, six of these resistant isolates also displayed cross-resistance to voriconazole.

### *Candida parapsilosis*

In the first study, *C. parapsilosis* was predominantly reported in premature neonates (28.8%) and patients with haematological malignancies (14.8%) [19]. Among these groups, *C. parapsilosis* infections were the leading cause of candidemia. With increasing patient age, the incidence of *C. parapsilosis* infection decreased from 28–33% to 6.9% [19].

In the second study, *C. parapsilosis* accounted for 18.5% of all cases [20], primarily affecting thoracic, neurosurgical, solid organ transplant and polytrauma patients [20].

Additionally, the species was associated with the colonization of catheters, neurosurgery, polytrauma, and infections in children under the age of 1 year [20]. The median time from ICU admission to the first positive blood culture was 16 days, with a mortality rate of 36.2% [20].

In the third study, *C. parapsilosis* was identified as the causative pathogen in 13% of all infections [21]. The emergence of fluconazole-resistant *C. parapsilosis* poses a major future risk for patients with candidemia. In the third study, fluconazole resistance was found in 17% of *C. parapsilosis* isolates, primarily from Greece, Italy, and Turkey. Echinocandin resistance was rare and detected in one isolate from Turkey, all resistance to both anidulafungin and micafungin and harbouring *fks* gene alteration. This echinocandin-resistant *C. parapsilosis* strain was also resistant to fluconazole, indicating multidrug resistance. Additionally, fluconazole and voriconazole cross-resistance was prevalent in *C. parapsilosis*, similar to *C. glabrata* (*N. glabratus*), albeit with differing geographical distributions [31].

### *Candida tropicalis*

*C. tropicalis* was consistently detected across all three studies, accounting for 7.2% of cases in the first study [19]. Notably, it was linked to the highest mortality rates (45%) [19]. In the second study it was isolated in 6% of all patients with invasive candida infections [20], with a crude mortality rate of 26.8% [20]. During the third study it was identified as the causative pathogen in 7% of all cases [21]. Beside other risk factors, the presence of *C. tropicalis* as causative pathogen emerged as an independent baseline predictor of candidemia mortality [21]. In the third study, fluconazole resistance was observed in 4% of *C. tropicalis* isolates, with varying distributions across Europe. Echinocandin resistance was rare, with no reported cases in *C. tropicalis* isolates. However, fluconazole resistance was detected in a small percentage of *C. tropicalis* isolates [31].

### *Candida krusei* (*P. kudriavzevii*)

In the initial study, *C. krusei* (*P. kudriavzevii*) was predominantly isolated from patients with haematological malignancies [19]. Subsequently, in the second study, this pathogen accounted for 2.5% of all invasive isolates. Of all the species isolated in the second study, it had the shortest median time to the first positive blood culture at 6 days, with an associated mortality rate of 57.9% [20]. The third study indicated that 3% of all infections were caused by *C. krusei* (*P. kudriavzevii*), representing a slight increase compared to the findings of the second study [20, 21].

### *Candida auris*

The species was only identified during the third ECMM study, *Candida* III, as a causative pathogen in 2.6% of cases [21] (Table 1).

**Table 1:** Species distribution *Candida* I to *Candida* III

Causative species	*Candida* I		*Candida* II		*Candida* III	
	N	(%)	N	(%)	N	(%)
*C. albicans*	1178	56.4	436	54.0	287	45.4
*C. glabrata (N. glabratus)*	284	13.6	111	13.8	133	21.0
*C. parapsilosis*	278	13.3	149	18.5	83	13.1
*C. tropicalis*	152	7.3	49	6.1	46	7.3
*C. krusei* (*P. kudriavzevii*)	40	1.9	20	2.5	16	2.5
*C. guilliermondii* (*Meyerozyma guilliermondii*)	30	1.4	5	0.6	6	0.9
*C. lusitaniae* (*Clavispora lusitaniae*)	15	0.7	14	1.7	6	0.9
*C. auris*	–	–	–	–	16	2.6
*C. kefyr* (*Kluyveromyces marxianus*)	10	0.5	2	0.2	5	0.8
*C. pelliculosa* (*Wickerhamomyces anomalus*)	9	0.4	3	0.4	2	0.3
*C. dubliniensis*	6	0.3	9	1.1	10	1.6
Other	51	2.4	22	2.7	11	1.7
Unidentified	9	0.4	–	–	13	2.1
Total	2089		807		632	

N = Number of isolates; Other: Candida I: *C. famata* (*Debaryomyces hansenii*) (n = 7), *C. lipolytica* (*Yarrowia lipolytica*) (n = 6), *C. norvegensis* (*Pichia norvegensis*) (n = 5), *C. inconspicua* (*Pichia cactophila*) (n = 4), *C. utilis* (n = 2), *C. sake* (n = 2) Candida II: *C. ciferrii* (n = 4), *C. haemoloni* (n = 2), *C. lambica* (*Pichia fermentans*) (n = 1), *C. humicola* (n = 1) Candida III: *C. norvegensis* (*Pichia norvegensis*) (n = 1), *C. digboenis* (n = 1), *C. rugosa* (n = 3), *C. inconspicua* (*Pichia cactophila*) (n = 2), *C. famata* (*Debaryomyces hansenii*) (n = 2)

## Risk Factors for the Development of Invasive *Candida* Infection

**Fig. 3:**
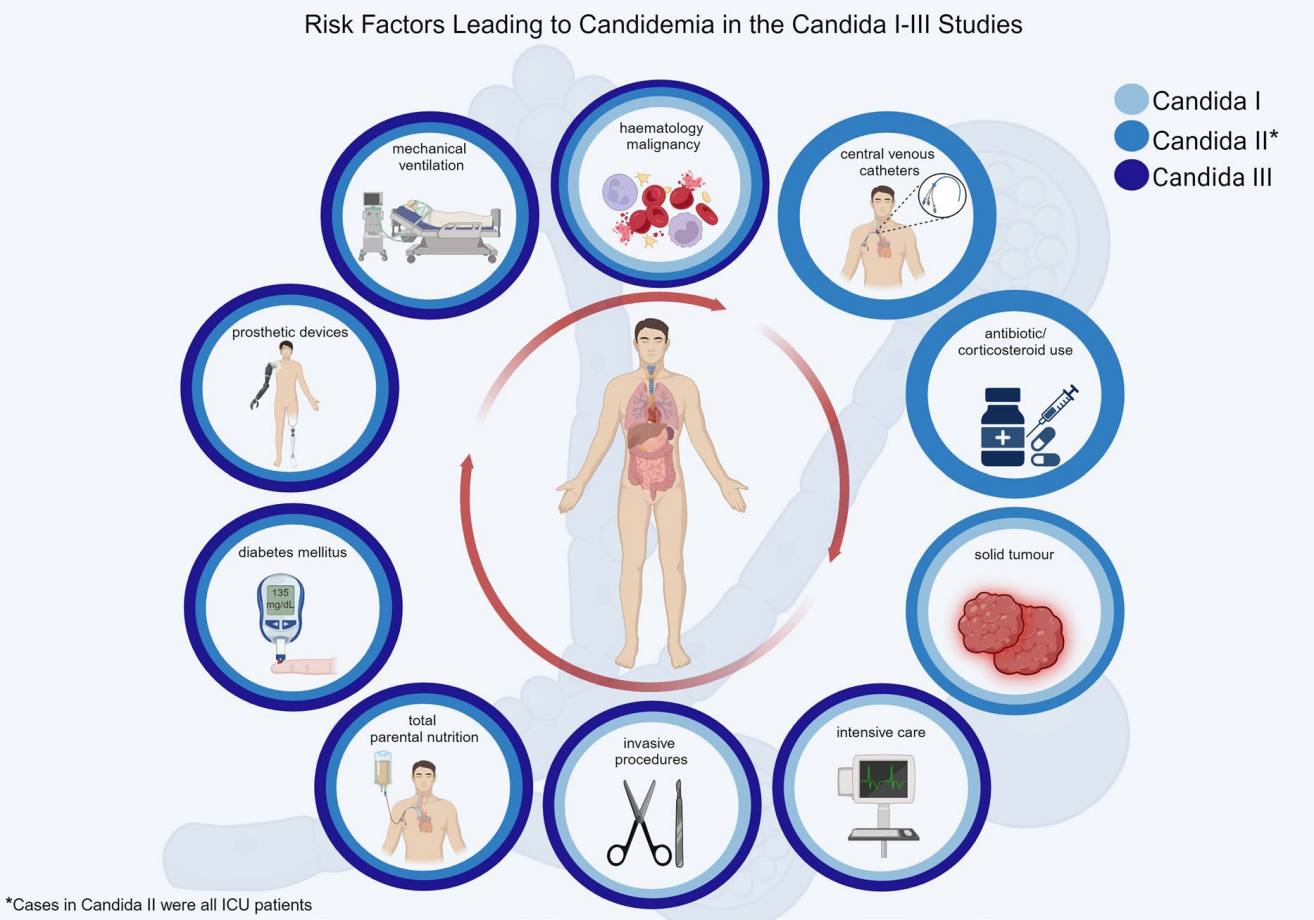
Summary of risk factors associated with candidemia development across three ECMM studies. The presence of risk factors observed during the studies is represented by blue circles (dark blue for Candida III, blue for Candida II and light blue for Candida I)

Several key risk factors and underlying diseases have been identified contributing to the development of invasive *Candida* infections in the three ECMM *Candida* studies (1997–2022) [19–21]. These factors include advanced age, major surgery, ICU admission, solid tumours, haematological and oncological malignancies, solid organ transplantation, corticosteroid use, total parenteral nutrition, use of CVCs and burns [19–21]. In the second study, which focused only on ICU patients, additional factors such as the use of broad-spectrum antibiotics in the previous 2 weeks (78.4%), rheumatologic disease (3%), steroid use in the previous 2 weeks (22.6%) and dialysis (18.2%) further contributed to the risk profile [20]. All risk factors that were present in all three ECMM studies are highlighted in Fig. 3 and listed in Table 2 (Supplementary files).

The majority of these risk factors were also identified by other studies [8, 36, 37]. Observations from all three studies, as well as other sources, highlighted that candidemia appears to be more prevalent in males across all age demographics [19–21, 38–40].

## Mortality

An alarmingly high mortality rate was observed in all three ECMM studies. The first study reported a crude mortality rate at day 30 of 37.9%, with highest rates for *C. glabrata* (*N. glabratus*) and *C. tropicalis* [19]. Increased mortality rates were particularly noted in patients of advanced age, those with cancer (especially with hematological malignancies) and patients in ICU. This underlines the influence of underlying medical conditions on mortality rates [19].

The second study yielded similar findings, with a crude mortality rate of 38.8% at day 30, with infants aged 3–12 months experiencing the highest mortality rates, reaching 72.7%. The highest crude mortality rates were observed with *C. glabrata* (*N. glabratus*) (43.6%) and *C. krusei* (*P. kudriavzevii*) *(*57.9%), followed by *C. albicans* (36.8%), *C. parapsilosis* (36.2%) and *C. tropicalis* (26.8%) [20]. In contrast to the first study, *C. tropicalis* and *C. parapsilosis* had low mortality rates during the second study [20]. This contrasts with findings from other studies as well [41]. Additional factors independently associated with high mortality included patient age over 60 years, the use of a CVC, administration of corticosteroids, absence of systemic antifungal treatment for IC, and failure to remove intravascular lines [20].

The latest study found an overall 90-day mortality of 43%, with 30-day mortality at 38% and 180-day mortality was 45% [21], aligning with the mortality rates described in earlier studies [19, 20, 42]. In contrast to the first two studies, ECMM *Candida* III included matched controls, enabling the calculation of attributable mortality, which was 18.1% [43]. Baseline predictors of mortality included increasing age, higher Charlson comorbidity index scores and ICU admission. Infection with *C. auris* and other rare *Candida* species also emerged as predictors of mortality [21]. The increase in non-*albicans Candida* species, coupled with the emergence of *C. auris* and increasing resistance rates, such as observed for fluconazole-resistance in *C. parapsilosis,* could pose significant risks for future candidemia patients [21].

As in the first two studies, independent baseline predictors of mortality were identified in the third study, such as older age and ICU admission. In addition, an increase in the Charlson Comorbidity Index, lower adherence to guideline recommendations and C. *tropicalis* as the causative pathogen were identified as predictors of mortality. Patients receiving an echinocandin as initial treatment (42%) had a lower overall mortality compared to patients without (52%) [21]. Additionally, the mortality rate for patients for whom guideline-recommended diagnostic or therapeutic measures were not performed was higher than in the overall cohort, emphasizing the importance of adhering to each guideline recommendation for the successful treatment of candidemia. [21].

## Antifungal Treatment

In the initial study, 84.5% of patients received antifungal therapy, with fluconazole (58.9%), amphotericin B (20%), and lipid-based amphotericin B (9.6%) being the primary treatments. Vascular catheters were removed in 61.4% of all patients. However, due to the limited documentation of only the initial therapy, a comparison of different antifungal therapies was not conducted during this study [19].

In the second study, focusing on ICU patients after major surgery [20], antifungal prophylaxis was administered to 16.5% of all patients [20]. Additionally, concomitant bacteraemia was observed in 4.9% of patients with candidemia. Antifungal drugs used for prophylaxis were fluconazole (78%), caspofungin (7.1%), voriconazole (3.2%), liposomal amphotericin B (5.6%), and amphotericin B deoxycholate (3.9%) and other drugs (2.2%). Among patients from *Candida* II, fluconazole was the most frequently used initial antifungal treatment, followed by caspofungin, lipid-based amphotericin B, and voriconazole [20]. Furthermore, in 70% of cases, the vascular catheter was removed or changed at diagnosis. [20]. The survival rate for patients in whom the catheter was removed was 68.1%, compared to 48.8% for those in whom the catheter was not removed [20].

In the third study, 16.5% of patients received antifungal prophylaxis, predominantly with fluconazole. Echinocandins were the initial treatment in 56% of patients, and those who received echinocandins as initial treatment (42%) had a lower overall mortality rate compared to patients receiving other antifungals (52%). Furthermore, the choice of initial echinocandin treatment was associated with a prolonged hospital stay solely for the completion of antifungal therapy, primarily due to the unavailability of echinocandins in oral form. [21].

## Limitations Across the Studies

Limitation across all three studies included incomplete questionnaires and missing data, therefore not all data were available from all cases included. Additionally, convenience samples were used, particularly in *Candida* I and *Candida* II, and results may therefore not be applicable across all European countries. The mycological analyses of the *Candida* strains were not performed in a single reference laboratory during the second study, potentially affecting the consistency and accuracy of the results [20]. Furthermore, haemodialysis, a known predictor factor of mortality, was not assessed in the second study due to missing data [20].

In the third study, higher EQUAL-*Candida* scores were observed in long-term survivors compared to patients with early fatal outcomes. This observation could potentially be influenced by immortal time bias, as there may have been insufficient time for implementing diagnostic and treatment recommendations [21]. Despite adjustments made (e.g. exclusion of all patients dying within 14 days of diagnosis in subanalyses), which all confirmed the associations between better adherence to guideline recommendations and survival, the influence of survival time on EQUAL scores cannot be entirely ruled out [21]. Moreover, the availability of fungal diagnostics, expert consultations, and access to antifungal drugs varies worldwide, especially in low-income and middle-income countries, limiting the generalisability of the findings [21]. Settings with better access to diagnostics and antifungals were likely overrepresented in all three ECMM *Candida* studies [21, 44].

## Outlook *Candida* IV

In line with the worldwide ECMM ISHAM guidelines and other ECMM initiatives [45, 46], the upcoming fourth multicentre ECMM study will expand its focus from Europe to the world. ECMM *Candida* IV is about to commence, focusing exclusively on *non-albicans Candida* species causing candidemia. Data will be collected from various hospitals worldwide to investigate antifungal resistance and tolerance, alongside their association with clinical outcomes. Furthermore, the study will assess antifungal susceptibility against novel antifungals in late-stage clinical development, as well as antifungal resistance among non*-albicans Candida* causing candidemia and the correlation of in vitro data, antifungal treatment response and patient survival rates*.* Given that the objectives differ from ECMM *Candida* III, ECMM *Candida* IV will have no restrictions in terms of how many hospitals per country can participate, nor will there be a maximum limit of cases that can be entered by each participating hospital. This approach aims to lower the barrier for participation, making ECMM *Candida* IV the first study of its kind conducted worldwide. Outreach efforts through social media platforms, conferences, and scientific meetings aim to encourage centers worldwide to initiate the onboarding process by completing site registration and information forms. These forms will undergo review and approval by study coordinators. Previous participating centres, as well as ECMM Global Guidelines contributors and ECMM fellow groups, are also being actively contacted and invited to participate in the study. The study is expected to enrol a total of 2,000 patients with non-*albicans* candidemia from institutions all over the world, including reference laboratories in all UN regions. The observation period spans from April 1st, 2024, to March 31st, 2025, with the initial data publication/presentation of results at scientific meetings expected in 2026. With a strong translational focus, ECMM *Candida* IV will combine clinical mycology and patient outcomes with insights from basic mycology research laboratories, particularly exploring the impact of antifungal tolerance on large-scale patient outcomes.

## Conclusion

The three pan-European multicentre studies conducted by the ECMM between 1997 and 2022 have provided invaluable insight into invasive *Candida* infections. They have highlighted the evolving trends in the spread of *Candida* species, the worrying increase in antifungal resistance and the high mortality rate associated with these infections. The studies identified increasing rates of antifungal resistance among *Candida* isolates, highlighting the need for the development of novel antifungal agents. Despite advancements in antifungal therapy, mortality rates associated with invasive *Candida* infections persist at high level, emphasizing the importance of early diagnosis and appropriate management strategies.

One of the most important findings across the three studies is the notable shift in causative *Candida* species, with increasing prevalence of non-*albicans Candida* species. Additionally, there is a notable increase in emerging species such as *C. auris*, which appear to benefit from climate change and warmer temperatures. The ECMM *Candida* studies identified also increasing rates of antifungal resistance among *Candida* isolates, highlighting the need for the development of novel antifungal agents. Several new antifungal drugs are currently in clinical development, with new mechanisms of action and improved activity against *Candida* species. This could address current limitations in treatment, such as the need for prolonged hospitalization and frequent administration of medication.

Newly approved antifungals like ibrexafungerp, rezafungin, oteseconazole and miltefosine [47] represent significant additions to the existing antifungal arsenal. Additionally, promising drugs like fosmanogepix are undergoing clinical testing [47–49]. The approval of these medications and the development of upcoming candidates expand treatment options, potentially enhancing efficacy through various mechanisms and administration routes. [49].

Despite advancements in antifungal therapies, the persistently high mortality rate underscores the urgent need for continued research and development in this field. The high mortality rates observed across the three studies display the devastating outcomes in patients with candidemia and invasive candidiasis in real life, which differ from clinical trial settings where patients are selected carefully, often excluding those at highest risk. Moreover, access to essential fungal infection diagnostics and treatments remains limited, particularly in low- and middle-income countries. However this limitation persists even in certain lower income countries within Europe [50]. The upcoming global ECMM *Candida* IV study, with its focus on non-*albicans Candida* species and the evaluation of susceptibility and tolerance to novel antifungals worldwide, presents a unique opportunity to deepen understanding of the epidemiology, resistance and outcomes of candidemia caused by non-*albicans Candida* species.

### Supplementary Information

Below is the link to the electronic supplementary material.

Supplementary file1 (DOCX 26 kb)
