# Supplementary material for: Insights from Three Pan-European Multicentre Studies on Invasive Candida Infections and Outlook to ECMM Candida IV
Source: Mycopathologia. 2024 Aug 1;189(4):70. doi: 10.1007/s11046-024-00871-0 (PMC11294264; doi:10.1007/s11046-024-00871-0)
Supplement: Supplementary file 1 — Supplementary file1 (DOCX 26 kb) [file 11046_2024_871_MOESM1_ESM.docx]

**Supplementary table**

**Table 2** The most common risk factors for the development of candidaemia from all three studies. The major surgical procedures in Candida II were broken down in detail in the study and include the following types of surgery: abdominal (51.5%), thoracic (20%), vascular (6.3%), neurosurgery (8.2%), orthopaedic (1.5%), multiple trauma (6.9%).

| **Risk factor** | ***Candida* I** | ***Candida* II** | ***Candida* III** |
| --- | --- | --- | --- |
| Major surgery | 44.7 % | 94.5% | 26% |
| ICU | 40.2% | 100% | 37% |
| Solid tumour | 22.5% | 22.9% | no data |
| Haematological malignancy | 12.3% | 3.3% | 39% |
| Solid organ transplant | 3.5% | 3.3% | 2% |
| Burns | 1.4% | 1.2% | no data |
| Diabetes mellitus | no data | 15.2% | 22% |
| Total parenteral nutrition | no data | 44.4% | 22% |
| Prosthetic devices | no data | 5.5% | 12% |
| Mechanical ventilation | no data | 60% | 29% |
